# P-248. Implementation of a National Environmental Program Services (EPS) Ultraviolet-C (UV-C) No-touch Room Disinfection Toolkit in the Veterans Health Administration

**DOI:** 10.1093/ofid/ofae631.452

**Published:** 2025-01-29

**Authors:** Cassie Cunningham Goedken, Kimberly Dukes, Stacey Hockett Sherlock, Daniel Suh, Bernardino Guerrero, Trina Zabarsky, Eli N Perencevich

**Affiliations:** Iowa City VA Medical Center, Iowa City, Iowa; Iowa City VA, Iowa City, Iowa; Iowa City VA Medical Center, Iowa City, Iowa; Iowa City VA Health Care System, Iowa City, Iowa; US Department of Veteran Affairs, Lexington, Kentucky; US Department of Veteran Affairs, Lexington, Kentucky; University of Iowa/Iowa City VAMC, Iowa City, Iowa

## Abstract

**Background:**

Environmental disinfection reduces the risk of hospital infections. Prior evidence from the Veterans Affairs (VA) healthcare system suggests that adding UV-C systems to manual disinfection processes significantly reduced nosocomial infection rates. Many VAs have added UV-C disinfection, but with wide variation in implementation, effectiveness, and use. To address this, we developed, piloted, and implemented a national VA UV-C toolkit.

**Methods:**

Our VA multicenter implementation science program project grant collaborated with VA Environmental Programs Service (EPS) to rapidly develop and implement a toolkit for VA facilities to establish and maintain UV-C processes that maximize efficacy. In our larger study, we interviewed Environmental Management Services (EMS) staff and identified barriers to UV-C (e.g., safety concerns, material degradation). From 5/23-9/23, we identified best practices and ways to address implementation challenges to create a mixed-methods data-driven national EPS UV-C toolkit. We incorporated our prior VA effectiveness research and concerns stakeholders raised in EPS meetings and interviews.

**Results:**

The toolkit includes sections that were deemed important guidance for UV-C use: 1) Ultraviolet Light, 2) Disinfection, 3) Benefits, 4) Process for Disinfection, 5) Quality Assurance/Validation, and 6) Frequently Asked Questions. It was piloted at 6 geographically dispersed VAs (9/23-10/23). Pilot feedback was positive and led to revisions for site variability (e.g., revised room prep steps allowing for instructions to be more universally representative of room layouts), UV-C use (e.g., scenarios when UV-C can be useful), and monitoring details (e.g., track frequency and location of use). EPS implemented the final toolkit in March 2024 on the VA national SharePoint, online, and as a printable PDF.

**Conclusion:**

Implementation work in collaboration with operational partners and partnered pilot testing was successful in rapidly developing and implementing a standardized UV-C toolkit across a large healthcare system. The toolkit guides EMS professionals in establishing and standardizing processes for UV-C disinfection to improve Veterans’ safety.

**Disclosures:**

**All Authors**: No reported disclosures

